# Supplementary material for: ProteinShader: illustrative rendering of macromolecules
Source: BMC Struct Biol. 2009 Mar 30;9:19. doi: 10.1186/1472-6807-9-19 (PMC2672931; doi:10.1186/1472-6807-9-19)
Supplement: Additional file 1 — ProteinShader program without source code. This compressed file contains the complete ProteinShader program including associated libraries, but no source code. A README.txt file gives an overview of the ProteinShader distribution, and the index.html file in the help subdirectory has directions on getting started with the program as well as a set of tutorials. [file 1472-6807-9-19-S1.zip › ProteinShader-beta-0_9_4-binary/help/api/org/proteinshader/structure/io/enums/RecordEnum.html]

RecordEnum (ProteinShader API)


|  |  |  |
| --- | --- | --- |
| **Overview** \| **Package** \| **Class** \| **Use** \| **Tree** \| **Deprecated** \| **Index** \| **Help** | |  |
| **PREV CLASS**   **NEXT CLASS** | **FRAMES**    **NO FRAMES**     **All Classes** |  |
| SUMMARY: NESTED \| ENUM CONSTANTS \| FIELD \| METHOD | DETAIL: ENUM CONSTANTS \| FIELD \| METHOD |  |


---


## org.proteinshader.structure.io.enums Enum RecordEnum

```
java.lang.Object
  java.lang.Enum<RecordEnum>
      org.proteinshader.structure.io.enums.RecordEnum
```

**All Implemented Interfaces:**: Serializable, Comparable<RecordEnum>

---

``` public enum RecordEnum extends Enum<RecordEnum> ```

This file provides an enumeration of the record types defined in the
Protein Data Bank Content Guide (Version 2.2, 20 Dec 1996).
